# Human Respiration Rate Measurement with High-Speed Digital Fringe Projection Technique

**DOI:** 10.3390/s23219000

**Published:** 2023-11-06

**Authors:** Anna Lena Lorenz, Song Zhang

**Affiliations:** 1 Institute of Biomedical Engineering, Karlsruher Institute of Technology, 76131 Karlsruhe, Germany; lena-lorenz1@web.de; 2 School of Mechanical Engineering, Purdue University, West Lafayette, IN 47907, USA

**Keywords:** Fringe Projection Profilometry, structured light, respiration monitoring

## Abstract

This paper proposes a non-contact continuous respiration monitoring method based on Fringe Projection Profilometry (FPP). This method aims to overcome the limitations of traditional intrusive techniques by providing continuous monitoring without interfering with normal breathing. The FPP sensor captures three-dimensional (3D) respiratory motion from the chest wall and abdomen, and the analysis algorithms extract respiratory parameters. The system achieved a high Signal-to-Noise Ratio (SNR) of 37 dB with an ideal sinusoidal respiration signal. Experimental results demonstrated that a mean correlation of 0.95 and a mean Root-Mean-Square Error (RMSE) of 0.11 breaths per minute (bpm) were achieved when comparing to a reference signal obtained from a spirometer.

## 1. Introduction

Vital signs, such as body temperature, pulse rate, blood pressure, and the Respiration Rate (RR), are used to monitor the clinical status of patients and are crucial in detecting medical problems as they represent the fundamental functions of the body [1]. The RR is an early indicator for clinical deterioration and is therefore an integral component of numerous early clinical warning scores [2,3,4,5]. Changes in the RR can predict conditions including respiratory failure [2], cardiac arrest [3,5], chronic heart failure [3], pulmonary embolism [3], weaning failure [3] or Intensive Care Unit (ICU) readmission [3]. Liu et al. [3] reported that deviations in the RR from its normal range can identify risks for medical emergencies 24 h before their occurrence with a probability of 95%. They also noted that the RR provides a better discriminator than blood pressure or heart rate in identifying high-risk patients. To measure the RR, the common practice involves manually counting the number of breaths within a specific time frame [3,6,7]. According to Drummond et al. [4], the measured RR can vary by 2 to 6 breaths per minute (bpm) between observers. Moreover, in busy clinical environments, there is often insufficient time to measure longer time frames for each patient and repeat measurements regularly. Consequently, shorter time intervals of 15 to 30 s are typically used, further contributing to the variance [4,7]. This variance can result in up to 40% of failures in the early warning scores due to inaccurate RR measurements [4].

Measuring a single RR value is crucial, but continuous monitoring of respiration over an extended period can be necessary in certain cases. For instance, respiration has been examined for diagnosing and monitoring patients with conditions like chronic obstructive pulmonary disease [6], pneumonia [3,5] or sleep breathing disorders [6]. Continuous respiration monitoring also finds application in diagnostic imaging and radiation therapy, where the identification and elimination of motion artifacts caused by patients breathing can enhance measurement quality [5]. While more accurate methods for measuring respiration signals, such as spirometry [6], plethysmography, capnography, piezoelectric and bioimpedance-based sensors [7] exist, these methods are often intrusive, interfere with the normal breathing or cause discomfort for patients. The current diagnosis of sleep breathing disorders relies on polysomnography, which is highly uncomfortable and often results in non-representative sleep [6]. In some cases, infants in the neonatal intensive care unit are too small to use the face masks of the traditional methods. Recently, there has been a growing awareness of the importance of respiratory monitoring for patients with viral infections, such as COVID-19 [2,5]. Minimal contact between clinical staff and the patient is needed. Consequently, contactless continuous monitoring systems have gained greater interest to address these challenges [5]. The aim is to reduce the restrictions and improve patient comfort [2] while simultaneously providing an automated, reliable and accurate solution [3]. Contactless systems differ in their underlying mechanisms and have different advantages and limitations [3]. Massaroni et al. [5] provided a comprehensive review of these contactless techniques. The contactless methods measure respiratory sounds, air humidity [8], air temperature, cardiac activity modulation or chest wall movements. To measure respiratory sounds in a contactless manner, environmental microphones are used. The advantage lies in its commercial availability, but challenges persist in distinguishing between inhaling and exhaling sounds and in its sensitivity to environmental noise [9]. Zhang et al. used electrochemical humidity sensors with a breathing mask to measure the respiration rate and detect respiration patterns [8]. Thermal cameras are used for the contactless measurement of air temperature, while red, green, and blue (RGB) cameras can capture cardiac activity. However, 2D imaging-based methods like RGB cameras or thermal cameras usually require the patients face to be visible, limiting their utility in regard to patient positions and ambient lighting conditions [10].

To measure the chest wall movements and extract respiration parameters, several technologies have been used, including marker-based systems [9], laser vibrometry [11], radiofrequency sensors [12,13], terahertz sensors [14], ultrasound [15], RGB cameras [16,17,18] and depth sensors [2]. While marker-based systems are classified as contactless, they still require markers to be attached to the body. Consequently, some of the disadvantages of contact-based methods persist, and these systems are in general expensive [5]. Hence, marker-based systems are often unsuitable for contactless continuous monitoring [5]. Although the physical principles differ among these sensors, they all rely on distance information between the body surface and the sensor. Depth sensors, also known as 3D sensors, can be based on the principle of Time-of-Flight (ToF) [10,19,20,21,22,23] and structured light [24,25,26,27]. Many studies focus solely on extracting the average RR, while some extract the Breath-to-Breath Respiration Rate (BRR), measure volume variations, or extract a continuous respiration signal. Among these 3D sensing technologies, ToF sensors have been predominantly used for monitoring breathing activity [5] due to the sensor’s commercial availability and low cost. The Microsoft Kinect v2 ToF sensor has been employed in multiple studies [19,21,28]. Skeletal information was automatically provided by the Kinect SDK 2.0. The Region of Interest (ROI) can be defined based on the joint positions of the shoulders, hips, and mid-spine. However, these systems are limited to capture subjects facing the camera, as the joints cannot be detected and the ROI computation fails otherwise. Kempfle et al. [19] and Aoki et al. [21] indicate that such a method is only suitable for non-moving subjects. To address this limitation, Kempfle et al. [28] detected and used the throat region, minimally affected by respiratory motion, as a reference for overall body motion estimation. This method enabled more accurate measurement of respiratory motion by removing the overall body motion from the signal. Their approach achieved a correlation to the signal from a respiratory belt of over 0.9 for more than half the users, even with body motion. However, when the throat region is covered due to head movements, changes in position, or occlusion, the correlation dropped below 0.4, restricting the feasibility the feasibility of the method for various patient positions. Gleichauf et al. [10] proposed the use of a ToF camera in combination with a microwave interferometric radar sensor, specifically for Neonatal Intensive Care Units (NICUs). The method was tested using a self developed neonatal simulation system for different breathing patterns. The ToF camera outperformed the radar sensor in the normal range of the RR but led to less accurate results in the low RR region, differing 31 bpm from the reference rate. The ToF camera’s strong sensitivity to noise and reflection caused the algorithm to detect additional peaks at low RRs. The radar sensor was found to be sensitive to body motion artifacts, such as swaying arms. By combining the two methods, they were able to extract an RR in the normal range with a maximum deviation of 3 bpm compared to the reference, by complementing each other’s drawbacks. Another 3D sensing technology, the structured light approach, was used in [24]. The system was specifically designed for individuals with a normal breathing rate. Different scenarios, including slight body movement, different clothing, ambient lighting conditions, and user orientations were compared. The system demonstrated correlations between 0.92 and 0.98 with the spirometer across these scenarios. However, the main limitation identified was the patient’s orientation, allowing only a maximum deviation of 25${}^{\circ }$ from the frontal view. Consequently, the system is restricted to a limited range of patient positions. The approach proposed in [25] was also based on structured light, using a pattern composed of RGB primary colors to illuminate the scene. The subjects were unclothed and laying still. While an interference effect from ambient light on measurement accuracy was observed, it was not quantified. Under the predefined conditions, they were able to generate motion trajectories of a specific point near the diaphragm. However, the results were not verified using a reference system.

In many studies, the selection of the ROI was manually conducted [20,23,29,30,31]. However, a recent study [32] focused on the automated selection of the ROI. This process involved dividing each depth frame into sub-regions, averaging the depth in each region, and then applying blind-source separation to each time signal. A scoring system was applied to assess the resulting signals based on their likelihood of originating from respiratory activity. As a reference, a respiration belt was used, with participants instructed to minimize body movement and breathe normally. On average, errors between 2.89 and 11.2 bpm were observed in the detected RR. It is important to note that the scoring system relies on characteristics of normal breathing and is not suitable for detecting abnormalities. Despite recent advancements in contactless respiration monitoring techniques, challenges and questions remain unsolved [7]. The primary challenges include the system’s robustness in varying ambient lighting conditions, body movement, patient positions, range of RRs, clothing and also the overall accuracy in general. Moreover, many studies focus solely on extracting the average RR rather than a continuous respiration signal.

In recent years, remarkable progress has been achieved in contactless 3D shape measurement technologies in general, which is driven by their increasing demand across various industries [33]. Those advances had, among others, been made in the field of Fringe Projection Profilometry (FPP). According to several authors [33,34,35], FPP is one of the most popular and promising technologies for 3D measurements. Systems based on FPP show advantages in its achievable speed, depth resolution, accuracy, affordability, versatility and robustness [33,36] in comparison to other 3D sensing technologies such as ToF cameras or stereo vision.

This paper introduces a FPP-based contactless respiration monitoring approach and its evaluation to overcome some of the challenges described above. The focus lies on the analyzing algorithm for extracting the RR and a respiration signal from the FPP 3D measurements. The system is evaluated in terms of its overall accuracy, robustness and limitations.

## 2. Materials and Methods

### 2.1. System Overview

**Figure 1:**
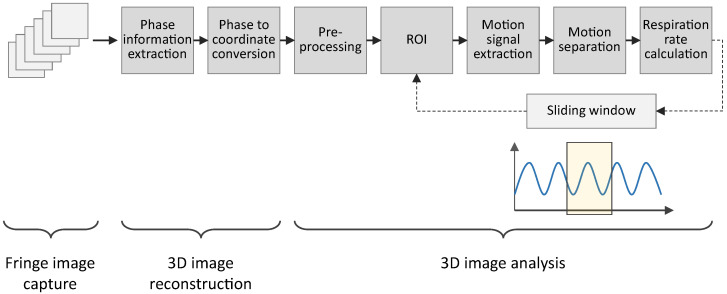
Overview of the developed system from capturing data to the extraction of the respiratory parameters.

The system developed in this project comprises the 3D sensor system and algorithms designed to extract respiratory parameters from 3D images. Figure 1 illustrates the data flow from the measurements to the extracted respiratory parameters. The 3D sensor system captures 2D images and reconstructs 3D images from those 2D images. The analysis of the 3D images involves six key steps for extracting the respiratory parameters.

### 2.2. Three-Dimensional (3D) Sensor System

To obtain a 3D representation of the thorax and abdomen, an FPP-based 3D sensor system was used. Due to its flexibility and versatility, a digital video projector was utilized for projecting computer-generated patterns. Specifically, a DLP LightCrafter 4500 projector was used, and its specifications are summarized in Table 1. The camera specifications can be found in Table 2. Additionally, an Arduino microcontroller was responsible for the synchronization between the camera and the projector.

**Figure 2:**
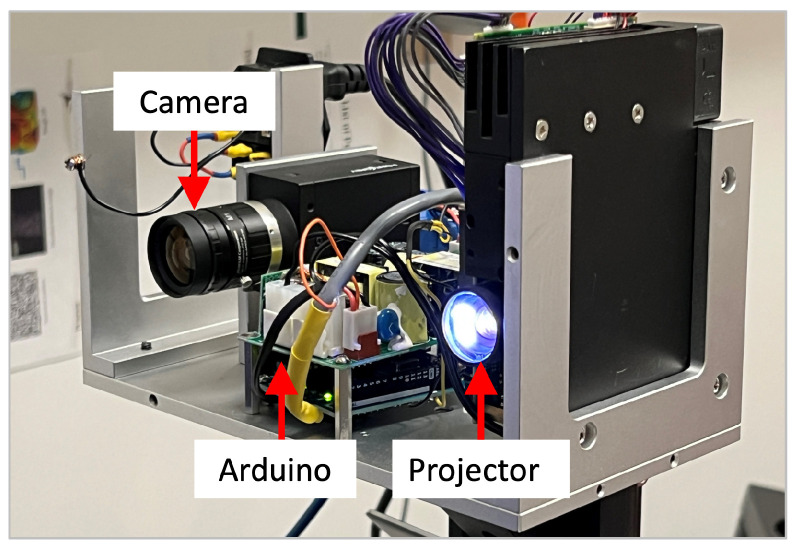
Setup of the 3D sensor prototype.

While the projector has a fixed resolution and a variable frame rate over 4 kHz [37], the camera’s resolution and frame rate are dependent. A higher frame rate can only be achieved by reducing the resolution of the camera [38]. Balancing speed and resolution led to a camera resolution of 640×480 pixels and a frame rate of 330 Hz, resulting in a sampling frequency of 55 Hz for 3D image capture. The corresponding ideal focal length of the lens was calculated as f=5.37 mm, ensuring an adequate field of view (FOV) encompassing the upper body. Considering the limited options for affordable machine vision lenses, a focal length of $f_{real}=6$ mm was chosen [39]. Opting for a longer focal length results in a smaller FOV of the camera, ensuring full coverage of the camera’s FOV by the projected area. The geometrical parameters of the 3D sensor are summarized in Table 3. Figure 2 shows the setup of the camera, the projector, and the Arduino.

While the fundamental concept remains consistent across all FPP methods, there are variations in the specific details, such as the fringe analysis method, fringe pattern design, and unwrapping method. In this study, the Phase-Shifting Method (PSM) was used as the fringe analysis method. According to Zhang [39], the PSM demonstrates reduced sensitivity to surface texture variations, while other methods are sensitive to noise and surface texture and/or geometry variations. Due to its high speed, accuracy, resolution, and robustness, the PSM has found extensive use in optical metrology [36], making it well suited for this system, particularly concerning spatial resolution and robustness.

To achieve a high speed, a three-step phase-shifting method is typically used. Three phase-shifted fringe patterns with equal phase shifts can be described as

(1) $$\begin{array}{ccc} I_{1} & = & I^{\prime }+I^{\prime \prime }\cos\left(\phi -2\pi /3\right), \end{array}$$

(2) $$\begin{array}{ccc} I_{1} & = & I^{\prime }+I^{\prime \prime }\cos\left(\phi \right), \end{array}$$

(3) $$\begin{array}{ccc} I_{1} & = & I^{\prime }+I^{\prime \prime }\cos\left(\phi +2\pi /3\right), \end{array}$$

where $I^{\prime }$ is the average intensity, $I^{\prime \prime }$ is the modulation, and ϕ is the phase to be solved. Simultaneously solving these three equations gives

(4) $$\phi =\tan^{-1}\frac{\sqrt{3}\left(I_{1}-I_{3}\right)}{2I_{2}-I_{1}-I_{3}}.$$

Due to the use of an inverse tangent function, the phase value extracted here is called the wrapped phase, whose values ranges from −π to +π. The phase has to be unwrapped to obtain a smooth phase map. Phase unwrapping essentially determines the integer number of 2π to be added for each pixel

(5) Φ=ϕ+κ×2π,

where Φ refers to the unwrapped map and κ denotes the integer number, which is often called the fringe order. In this research, we employed the enhanced two-frequency phase-unwrapping method developed by Hyun et al. [40]. For such an algorithm, six fringe images were utilized, consisting of two different fringe periods: $\lambda_{1}=30$ pixels for the initial three images and $\lambda_{2}=380$ pixels for the subsequent three images [40]. Additionally, the binary defocusing technique proposed by Li et al. [41] was employed to allow the projector to operate at significantly higher frequencies, thereby enabling high temporal resolution. Using fringe patterns containing multiple periodic stripes necessitates the unwrapping of the phase obtained from the PSM [39].

The calibration of the 3D sensor system was essential to determining both the extrinsic and intrinsic parameters of the system using the standard pinhole model for both the projector and the camera. The pinhole model describes the relationship between the 2D coordinates of the sensor pixel and the corresponding 3D world coordinates (x,y,z),

(6) $$\begin{array}{ccc} s^{c}{\left[u^{c},v^{c},1\right]}^{T} & = & A^{c}\cdot \left[R^{c},t^{c}\right]\cdot {\left[x,y,z,1\right]}^{T}, \end{array}$$

(7) $$\begin{array}{ccc} s^{p}{\left[u^{p},v^{c},1\right]}^{T} & = & A^{p}\cdot \left[R^{p},t^{p}\right]\cdot {\left[x,y,z,1\right]}^{T}, \end{array}$$

where ${}^{c}$ denotes camera, ${}^{p}$ denotes projector, $s^{c}$ and $s^{p}$ denote the scaling factors, $A^{c}$ and $A^{p}$ denote the 3×3 intrinsic matrices, $R^{c}$ and $R^{p}$ denote the 3×3 rotation matrices, $t^{c}$ and $t^{p}$ denote the 3×1 translation vectors, and ${}^{T}$ denotes the matrix/vector transpose operation. For this purpose, the calibration method outlined in [34] was adopted. Once the system is calibrated, those matrices are known. Equations (6) and (7) have 6 equations with 7 unknowns $x,y,z,s^{c},s^{p},u^{p},v^{p}$ for any given camera image pixel $\left(u^{c},v^{c}\right)$. The unwrapped phase Φ of that pixel provides another constraint equation that allows us to solve (x,y,z) coordinates uniquely pixel by pixel to reconstruct the 3D shape of the object.

### 2.3. Three-Dimensional (3D) Image Analysis

The resulting 3D point data, captured by the 3D sensor described above, were analyzed to extract a respiration signal, an RR and a BRR from the chest wall movement. Our methodology placed significant emphasis on ensuring the system’s robustness and the generation of continuous results. Consequently, potential patient movements, variations in patient positions, and diverse surface textures were considered in the development. The processing involved six main steps, as depicted in Figure 1, each of which is elaborated upon in the subsequent sections.

#### 2.3.1. Step 1: Preprocessing

The captured fringe images undergo preprocessing before the reconstruction of the 3D coordinates. A 5×5 Gaussian filter, with a standard deviation of 1.67, is applied to reduce significant noise. Subsequently, a threshold is applied to the intensity modulation of the captured sinusoidal patterns to filter out low-quality phase points.

**Figure 3:**
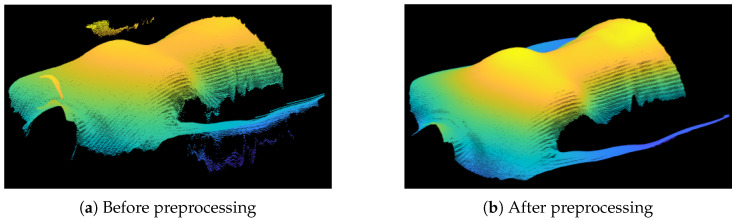
Comparison between two point clouds with and without preprocessing. Color represents the distance from the sensor with light gold points being closer dark blue points being further away.

The captured intensity modulation varies depending on the surface properties. The threshold aids in distinguishing high-quality and low-quality image points. To build a robust system, the threshold dynamically adjusts based on the overall light intensity in the image. Consequently, the system can adapt to varying object surfaces, particularly those with varying colors. After the 3D coordinates are calculated and saved as a matrix of (x,y,z) coordinates, additional preprocessing steps were implemented. As depicted in Figure 3a, the 3D structure comprises the desired torso coordinates along with invalid outliers and parts of the background. Depth sensors relying on light reflection have often larger measurement errors in surface areas with steep angles toward the sensor [19], leading to outliers, especially in the body’s marginal regions. The removal of outliers involves calculating the derivative of the *z* coordinate along each row and column. A threshold is then applied to eliminate pixels displaying rapidly changes in geometry compared to their neighboring pixels. Subsequently, isolated pixel clusters containing a minimal number of points are removed, aiming to eliminate points not belonging to the main body. The final preprocessing step involves the application of a median filter [19] to further reduce noise in the captured surface. All points filtered out through these preprocessing procedures are designated as Not a Number (NaN) values. Figure 3b illustrates the cleaned 3D data.

#### 2.3.2. Step 2: Determination of Region of Interest (ROI)

**Figure 4:**
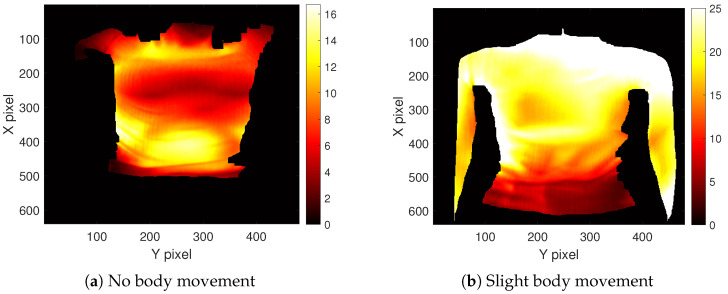
Maximum variation in the *z* direction in each pixel over a time frame of 30 s.

The selection of the ROI enables a focus on the body parts affected by respiratory movement. Lung volumes directly correlate with the expansion of the chest and abdomen. Depending on the type of breathing, predominant movement occurs either in the abdominal or thoracic region. Consequently, both regions are relevant for monitoring respiration [42]. Kempfle et al. [19] indicated that the two parameters, namely the size and position of the ROI, significantly influence the outcome. A smaller ROI is more sensitive to noise, while an excessively large ROI may capture additional body parts that negatively impact the measurement. The ROI’s position has to cover the areas where the respiratory motion is noticeable. Representative heat maps illustrating the maximum variation in the depth value of body regions during respiration are depicted in Figure 4a,b. Figure 4a displays the outcome when the subject was instricted not to move with the arms positioned outside the camera’s FOV. In contrast, Figure 4b depicts a scenario with the arms within the FOV, and the subject was permitted slight movement. Noticeable high variations are observed in the marginal area and around the arms due to these slight body movements, emphasizing the necessity of an ROI to ensure robustness. These observations verify the assumption of noticeable respiratory motion in the chest and abdominal areas. In the first case (Figure 4a), the variations are more severe in the abdominal region, while in the second case (Figure 4b), the variations in the chest region appear more significant. Therefore, both regions were considered in this study.

**Figure 5:**
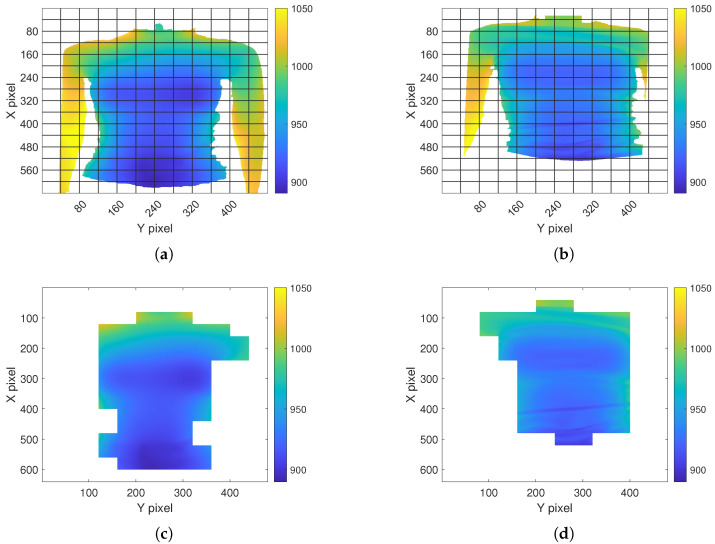
Dynamic adaption of the ROI selection before and after body movement. (**a**) Depth map of the body centered before movement. (**b**) Depth map of the body after movement. (**c**) ROI before movement. (**d**) ROI after movement.

Furthermore, subjects might reposition themselves within the camera’s FOV during the course of measurement. Consequently, the ROI needs to be adjusted accordingly. In numerous projects, the position and size of the ROI were manually determined post-recording [23,29,30,31]. However, such a manual approach lacks the capability to dynamically adapt the ROI in real time and requires the offline manual analysis. To address these limitations, the 3D image is segmented into voxels of 40×40 pixels, as illustrated in Figure 5a [17,32]. Each voxel is assigned a score distinguishing between areas within the ROI and excluded regions. Voxels containing invalid points (NaN values), represented as white areas in Figure 5a, are discarded. To enhance robustness, a voxel is considered only if it solely contains valid points in both the current and previous frame. As depicted in the example in Figure 5c, marginal areas affected by high variations and noise are not incorporated into the defined ROI. When the subject’s position changes, the ROI adjusts automatically. The proposed approach aims to automate the selection and dynamic adaptation of the ROI parameters’ position and size.

#### 2.3.3. Step 3: Extraction of Motion Signal

To derive the respiratory pattern from the 3D reconstructed points of the upper body, the movements of the chest and abdominal wall were utilized. Two general methods of depth sensors are found in the literature: one approach estimates the volume of the ROIs, while the other directly uses the distance from the body to the sensor. The estimation of the ROIs volume [20,21,22] presumes that the ROI remains constant [19]. Since depth sensors solely capture the body surface, the volume has to be approximated. Altering the size and position of the ROI also changes the volume even without respiratory motion. Thus, this approach is deemed unsuitable if the ROI is dynamically adapted. Consequently, we opted for the latter approach to estimate the motion signal.

Three main criteria were considered to accurately extract a motion signal: (1) the noise in the resulting motion signal should be as low as possible, (2) the algorithm should be robust toward a dynamically changing ROI, and (3) the duration of the computation should be low for continuous monitoring applications. The approach proposed in [24] was chosen and adapted to the FPP sensor used in this project.

The average depth D(k) is calculated as

(8) $$D\left(k\right)=\frac{1}{N}\sum_{i=1}^{N}z_{i}\left(k\right),$$

and then the weighted average W(k) is computed as,

(9) $$W\left(k\right)=\frac{\sum_{i=0}^{3}w_{k-i}D\left(k-i\right)}{\sum_{i=0}^{3}w_{k-i}}.$$

A sliding window is applied to four successive frames and moved one frame at a time. The weights are chosen as $w_{k}=1$, $w_{k-1}=0.7$, $w_{k-2}=0.4$ and $w_{k-3}=0.1$ [24]. Combining with the sampling frequency $F_{s}$, the derivative of the weighted average dW(k) is calculated,

(10) $$dW\left(k\right)=F_{s}\left(W\left(k\right)-W\left(k-1\right)\right).$$

The motion signal S(k) is calculated by summing up the mean values of the change over time as

(11) $$S\left(k\right)=\sum_{i=1}^{k}dW\left(i\right).$$

### 2.4. Step 4: Monitoring with Sliding Window

In the context of utilizing the system for monitoring purposes, it is essential to regularly update the respiration pattern during the measurement. Therefore, the complete signal cannot be processed all at once, and a sliding window approach is adopted instead. This approach entails a temporal buffer of size *W* that holds the last *W* raw signal values. To ensure that the window covers a full respiratory cycle, a minimal RR of $f_{resp,min}=6$ bpm is used [43]. The window size can subsequently be calculated as [17]

(12) $$W=60\times \frac{f_{sampling}}{f_{resp,min}}.$$

#### 2.4.1. Step 5: Motion Separation

The extracted motion signal includes not only the respiratory motion but also incorporates body motion, which could potentially generate motion artifacts. To enhance the system’s robustness, the motion signal undergoes further processing. This study proposes a motion artifact detection algorithm to detect and eliminate motion artifacts, comprising three essential steps. Firstly, an operator characterizing the motion is applied. Subsequently, a threshold is determined to differentiate between respiratory and body motion. Finally, a binary trust status is assigned to distinguish between valid signal components and invalid signal components rendered unusable due to significant body motion.

The operator used is the Taeger–Kaiser energy (TKE) operator defined for discrete time series S(k) as [44]

(13) $$\psi \left[S\left(k\right)\right]=S^{2}\left(k\right)-\left(S\left(k-1\right)S\left(k+1\right)\right).$$

In [45], the TKE operator was used to detect motion artifacts in the signal of an accelerometer. Since the motion signal described in Section 2.3.3 is a discrete time series, the TKE operator was adapted to this system for motion artifact detection.

This operator responds to both frequency and signal amplitude [45]. When the TKE value exceeds a certain threshold, the corresponding signal values are eliminated and augmented by interpolation. Considering that the TKE value integrates three consecutive motion signal values, all three values are eliminated once the threshold is exceeded. The threshold must be low enough to effectively detect motion artifacts yet high enough to avoid categorizing rapid breathing as a motion artifact. Instead of using a fixed threshold, it is dynamically adjusted to enhance the system’s robustness. One characteristic that distinguishes respiratory motion from other body motions is its periodicity. The auto-correlation is calculated to characterize this periodicity. In cases where primarily respiratory motion is present, the secondary maxima of the autocorrelation are high and located at the distance of two respiratory motion peaks. The current frequency of respiration can be estimated from this distance, thereby determining the threshold for the TKE operator as a function of the frequency. If motion artifacts are present in the window, explicit secondary maxima may be absent, or the autocorrelation value in the secondary maxima might be low. In such instances, the estimated frequency remains unchanged, and the threshold for the TKE operator remains constant. The threshold is recalculated not each time the window is shifted but rather after the window has been entirely moved by its size. Lastly, the binary trust status is established based on the TKE operator and autocorrelation. If the sum of the TKE values in the window exceeds a certain threshold value, the binary trust status is set to zero (low); otherwise, it is set to one (high). As discussed earlier, the TKE is dependent on frequency. The periodicity of the signal obtained from the autocorrelation is used to correct a falsely low trust status particularly in signals of high frequency. This compensates for minor body motions while labeling signal segments affected by long and intense body motions as invalid. The proposed approach combines the local TKE operator with the global correlation across the entire window, enabling the identification and compensation of locally corrupted signal segments within the context of the current breathing mode.

When a patient changes position during the measurement, not only can motion artifacts occur during the movement, but the body can also be closer or further away from the 3D sensor. To compensate the shift, the mean of the signal is calculated for each window and then subtracted from the signal values.

In addition to motion artifacts, the sensor noise can also affect the extracted motion signal. Large noise impact is alleviated by the approach described above, but a moving average filter of size 5 is applied to further reduce noise impact.

#### 2.4.2. Step 6: Calculation of Respiration Rate (RR)

From the processed signal, the RR can be derived, with the literature primarily suggesting two options for RR calculation. The first option involves calculating the frequency spectrum of the signal and identifying the frequency of the highest peak in the spectrum [16]. Alternatively, peaks in the time domain can be calculated and counted within a specific time frame [10,20,24]. The latter approach was chosen due to its computational efficiency and the ease of extracting the average RR from the window.

The MATLAB function “findpeaks” is employed to identify the peaks in the time domain. The threshold value of the peak detection function is dynamically adjusted based on the current respiration amplitude. The respiration amplitude is estimated from the current maximum and minimum values in the window and updated only when the binary trust status is high.

The time differences between successive peaks represent the respiratory periods [20]. To calculate the RR in bpm, the reciprocals of the respiration periods are multiplied by 60 s, resulting in a BRR [20]. To determine the average RR, the peaks are counted within a 30 s time frame. Dividing the number of breaths by the total duration of all breaths and multiplying by 60 yields the average RR. Signal segments marked with a low trust status are excluded from the RR determination with the average RR and BRR remaining at the last valid value in such cases.

### 2.5. System Evaluation

#### 2.5.1. Reference System

To assess the system’s performance, a spirometer served as the reference system [2,5]. The spirometer enables a continuous and accurate measurement of lung volume variations [3]. As proposed in [46], the spirometer from the company “Vernier Software & Technology” was used, with the specifications summarized in Table 4 [47]. Through the Vernier’s Graphical Analysis App interface, the differential pressure (Pa) can be recorded over time. Additionally, the flow rate (L/s) and the tidal volume (L) can be derived. It is important to note that only the differential pressure is directly measured with the other categories being derived from this measurement. The flow rate is calculated using a calibration equation, and the tidal volume is obtained by integrating the flow rate [47]. Considering that chest expansion correlates directly with the inhaled and exhaled air volume, the tidal volume was used as the reference respiration signal.

**Figure 6:**
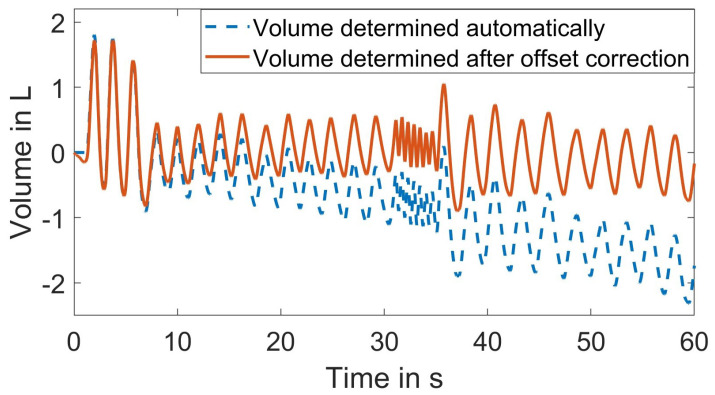
Comparison of the volume signal before and after baseline correction.

Figure 6 shows that an inherent baseline drift is introduced due to the internal volume calculation. To address this known issue, the volume can be automatically adjusted in the user interface [47]. The adjusted volume resets to zero after each ventilation cycle, effectively eliminating the baseline drift. However, this corrective measure distorts the shape of the volume signal. By eliminating the offset in the flow signal and integrating the corrected flow, the baseline drift in the volume signal can be minimized as shown in Figure 6. Given that the spirometer volume signal was used for comparing the pattern of the extracted respiration signal rather than absolute volumes, it was feasible to eliminate the linear trend in the reference signal. Furthermore, both the reference signal from the spirometer and the extracted respiration signal were normalized. To synchronize these two signals, the subject was instructed to breathe deeply three times at the beginning of the measurement.

To obtain a reference RR from the continuous volume signal, the volume peaks were detected and counted in a time frame of 30 s. To obtain a reference BRR, the time shift between two peaks was calculated as the respiration period. Its reciprocal multiplied by 60 yielded the BRR.

#### 2.5.2. Evaluation Metrics

The extracted respiration signal and the derived RRs were then compared to the reference system. To quantify the similarity between the extracted respiration signal and the reference signal from the spirometer, the cross-correlation between the two signals was computed. The time lag between the two signals was determined from the position of maximal correlation with a compensatory maximum shift of 0.27 s to accommodate minor time discrepancies in their synchronization. The Pearson correlation coefficient was then utilized as a measure of similarity [48]. The RRs were evaluated by calculating the Root-Mean-Square Error (RMSE) between the reference and the extracted RRs [20]. Both the RR and the BRR were assessed using this metric. Additionally, the results of multiple subjects and measurement series were consolidated by calculating the mean values and standard deviations.

To evaluate the system’s robustness and potential influencing factors, various scenarios were tested, involving different conditions and patient actions. The experiments included seven individuals, with each measurement lasting approximately 60 s. Since the first three breaths were solely for synchronization purposes, the usable signal was shorter. As a baseline, the subjects were instructed to lie in a supine position. The 3D sensor system was positioned above and pointing down toward the chest and abdomen, maintaining a calculated distance of 929.6 mm from the sensor to the subject. Accounting for the chest depth, the mean value between the minimum value and the maximum value from the literature was calculated as 236 mm, resulting in a rounded distance of 1165 mm between the sensor and the surface. Ambient light was turned on, and the subjects were wearing a white shirt. They were instructed to lie as still as possible and breathe naturally without conscious thought.

## 3. Results

### 3.1. Individual Processing Step Evaluation

As previously discussed, each 3D frame undergoes preprocessing before any further analysis. Firstly, we experimentally evaluated the performance of the preprocessing algorithm. Figure 3a,b depict an example 3D frame before and after applying our proposed preprocessing algorithm outlined in Section 2.3.1. Figure 3a illustrates the 3D point cloud without applying the preprocessing algorithm, while Figure 3b displays the 3D point cloud after preprocessing. The removal of invalid outliers and the smoother surface are evident. The remaining stripes, visible even after preprocessing, are due to the texture of the shirt that the subject was wearing. The depth (*z*) of each 3D frame was used for further analysis. The submitted Video S1 demonstrates a series of raw depth images before and after preprocessing, providing clear evidence of the effectiveness of the preprocessing algorithm.

**Figure 7:**
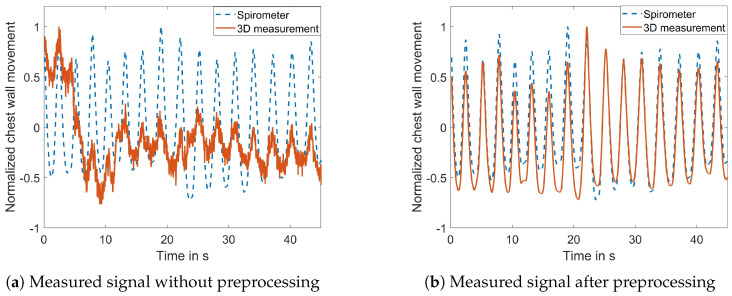
Comparison of the measured signal and the reference signal with and without preprocessing.

The impact of the preprocessing algorithm was further quantified by comparing the extracted motion signal to the spirometer signal. Figure 7a,b illustrate the normalized amplitude of the motion measured by the proposed 3D system in comparison to the spirometer’s result. Figure 7a depicts the motion signal extracted without preprocessing, displaying noticeable noise and a sudden jump at approximately 5 s. In contrast, Figure 7b shows reduced noise and the compensated jump after preprocessing. The correlation coefficient between the measured signal and the reference signal is 0.285 without preprocessing and 0.958 with preprocessing.

We then evaluated the effectiveness of the proposed dynamic ROI selection method. Figure 5 provides an example frame, while Figure 5a illustrates the 3D image when the body is centered in the FOV, from which the ROI is determined, as shown in Figure 5c. As the subject moved toward the right side of the FOV, the corresponding ROI also adjusted accordingly, as shown in Figure 5b,d. The submitted Video S2 demonstrates the adaptability of the proposed dynamic ROI selection algorithm in response to body movements. In contrast, Video S3 shows the usage of a fixed ROI.

**Figure 8:**
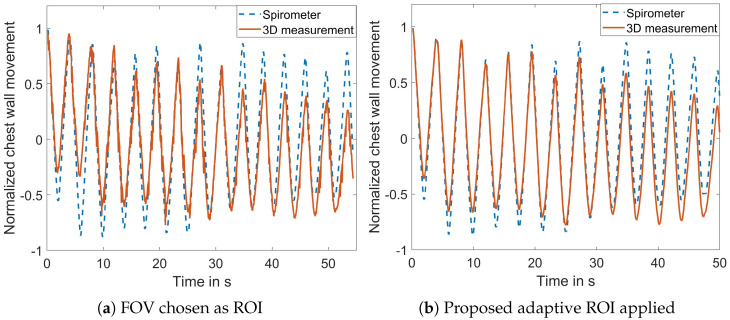
Comparison of the measured signal and the reference signal with and without the adaptive ROI selection.

The performance of the adaptive ROI selection algorithm was further quantified. Figure 8a diplays the signal when the entire FOV was selected as the ROI with the correlation coefficient between the extracted motion signal and the reference signal measuring 0.888. Upon applying the proposed ROI selection algorithm, the correlation coefficient increased to 0.955, as depicted in Figure 8b. This experiment further demonstrates the effectiveness of the proposed ROI algorithm to reduce the noise in the measured signal.

**Figure 9:**
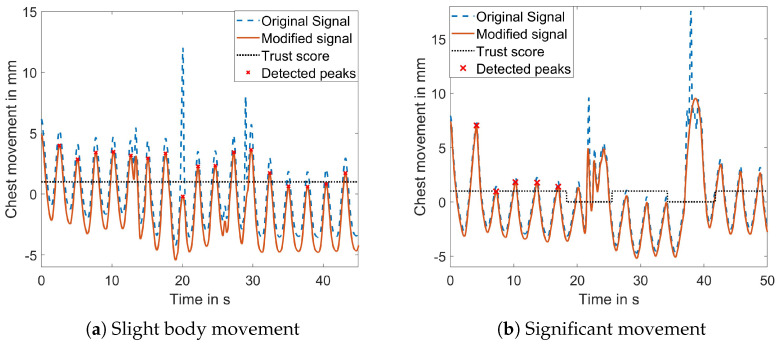
Result of the motion separation algorithm and the introduced binary trust status with different levels of body movements.

The motion separation approach was assessed by instructing the subject to move during the measurement. Two different levels of movement were defined: slight movement involving shoulder movement and changes in position and significant movement encompassing all degrees of freedom. Figure 9a illustrates a signal with slight body movement, demonstrating the successful detection and suppression of motion artifacts. The binary trust value remained high throughout the signal, which is desired because the binary trust value is important for the RR calculation, and if the motion artifacts can be compensated, the RR can be calculated. Additionally, the figure illustrates the successful detection of all respiration peaks.

If the body movement is more severe, too many points are removed from the signal, making it impossible to reconstruct the original course of respiration. In such cases, the binary trust status becomes crucial in preventing the misclassification of motion artifacts as respiration peaks. Figure 9b displays a signal affected by significant body movement, where the binary trust status marks the areas affected by the body movement. No peaks were detected in these areas for the RR calculation.

**Figure 10:**
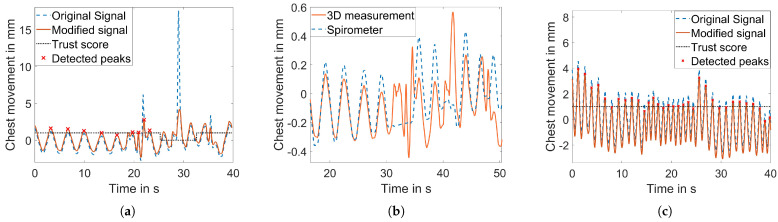
Result of the motion separation algorithm for three different breathing patterns and subject behavior. (**a**) Shallow breathing with slight body movement. (**b**) Holding the breath with body movement. (**c**) Fast breathing.

While the approach reliably provided a binary trust status in most cases, the limitation of the algorithm was found in the measurement shown in Figure 10a. When the breathing amplitude was shallower (1.2 mm in this case) and slight movement was present, the algorithm could not reliably differentiate between respiratory movement and motion artifacts. Consequently, the motion was not detected, and the body motion peaks were falsely identified as respiration peaks (Figure 10a). Figure 10b shows the second issue. At about the 30th second, the subject held the breath but started moving at the same time, and the algorithm failed to distinguish. Note that the amplitudes are normalized to compare the respiration signal to the spirometer signal. The main challenge was to detect the motion artifacts as abrupt changing signal parts but not to incorrectly classify fast breathing as motion artifacts. Figure 10c shows that the binary trust status was defined in a way that the fast breathing was not misclassified.

### 3.2. Overall System Performance Evaluation

The overall system performance and its robustness were assessed by the evaluation metrics described above under different scenarios that are summarized in Table 5. A baseline scenario was defined, and in each of the following scenarios, only one aspect was altered.

**Figure 11:**
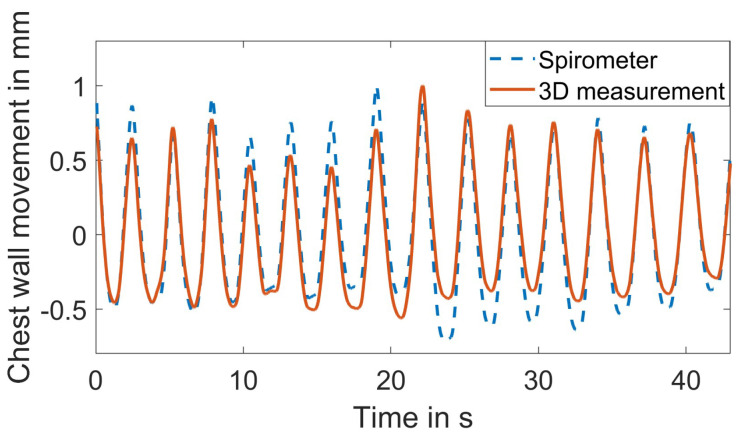
Comparison of the extracted respiration signal and the reference.

Figure 11 shows a representative result of the extracted respiration signal and the reference spirometer signal for the baseline scenario. The mean of all observed correlation coefficients in the baseline scenario is $\mu_{cc}=0.974$, and the standard deviation is $\sigma_{cc}=0.016$. Penne et al. suggested that correlation values between 0.69 and 0.87 are typical for systems currently utilized in clinical applications [30]. The mean correlation of $\mu_{cc}=0.974$ therefore confirms its clinical relevance.

**Figure 12:**
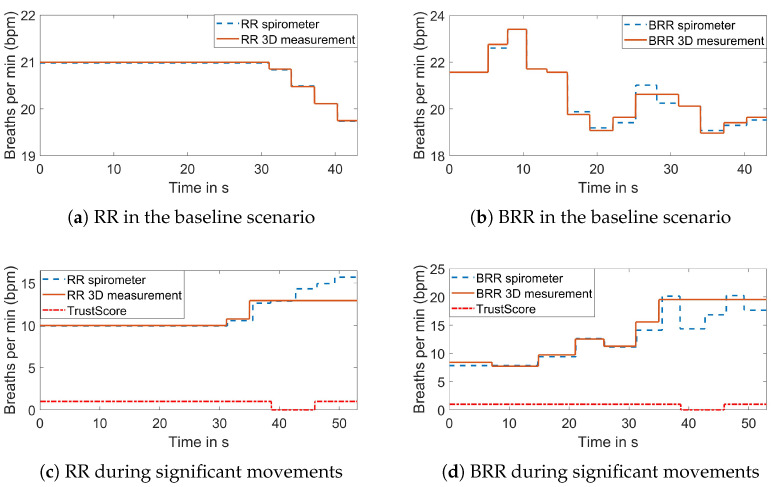
Comparison of the 3D measured RR and BRR and its reference.

Figure 12 illustrates the comparison of the average RR and BRR to the reference in the baseline scenario and during significant movements. In the baseline scenario, the mean error of the RR RMSE is $\mu_{RR}=0.048$ bpm, and the standard deviation is $\sigma_{RR}=0.027$ bpm. For the BRR, the mean error of the RMSE is $\mu_{BRR}=0.236$ bpm with a standard deviation of $\sigma_{BRR}=0.136$ bpm. In general, the BRR errors appear to be higher than the average RR. This is likely due to the BRR calculation being based on the individual respiration periods, and thus, a small deviation has a bigger impact on the BRR value. The assessment of the robustness toward body movements was divided into two parts. The first part was described above and focused on the motion detection. In this paragraph, the focus is on the overall performance with regard to body movement. While the removed motion artifacts from slight body movements could be reconstructed leading to a correlation coefficient higher than 0.85, significant movements resulted in a much lower correlation coefficient. This indicates that the reconstruction with interpolation was only sufficient for slight body movements. The errors were high in the RMSE values of the RR and the BRR for significant movements. Investigating the individual measurements in the significant movement category showed that the errors occurred primarily when the signal was not trusted and the RR and BRR values were kept at the same level. Figure 12c,d show an example of the resulting RR and BRR. After approximately 35 s, the RR and BRR were held at their current values, but in reality, the RR and BRR changed. It can be seen that the area of the low trust status is narrower than the area where the values are constant. Since the last reliable value is used, the constant part starts earlier. After the binary trust status is changed to high, a new window is needed to update the values. The remaining window at the end was too small to do so. Depending on how early or late the movement occurred in the signal and how severe the breathing changed during movement, the overall RMSE value was larger or smaller, leading to larger standard deviation values.

The different scenarios were assessed on several subjects that were part of this study. To summarize the results of the different subjects for each scenario, the mean and standard deviation were calculated for each measure and scenario and are shown in Table 6.

The respiration mode was assessed in terms of alternating fast breathing, deep thoracic or chest breathing, deep diaphragm or abdominal breathing and holding the breath. In all cases, the correlation coefficients are similar to the baseline results, showing no clear trend between the categories. Comparing the results in terms of the RMSE of the BRR, the fast breathing mode showed a slightly higher mean RMSE compared to the baseline results. This discrepancy is probably caused by the calculation of the BRR. Assuming a minimal RR of 6 bpm implies that the respiration periods have an average length of 10 s. Varying the respiration periods by 0.1 s between the measurement and the reference results in an error of 0.06 bpm in the BRR. On the other hand, assuming a maximum RR of 60 bpm leads to respiration periods of 1 s. In this case, a variation of 0.1 s in the respiration periods between the measurement and the reference causes a deviation of 5.5 bpm, which is significantly higher. Consequently, the same respiration peak detection accuracy algorithm leads to higher BRR errors during fast breathing due to its relatively larger error range. Regarding deep abdominal and chest breathing, the higher standard deviation is noticeable. This is likely due to body size and physique having a greater influence when breathing in deeply. However, in all categories, the mean RMSE of the BRR remained small: below 1 bpm.

Three different positions were assessed: the baseline position where the subject was lying on its back, the prone position and the lateral position. The mean correlation was high in all positions. The errors in the calculated RR and BRR were lower in the prone position and higher in the lateral position. This discrepancy is likely due to the resulting signal being noisier in the lateral position, which is attributable to the increased degrees of freedom for body movements.

The performance of the proposed method was also evaluated under three scenarios: varying shirt colors, no ambient light, and the use of a blanket. The correlation coefficient was consistently above 0.85, and the mean of the RMSE of the RR was low in all scenarios. Notably, the RMSE value of BRR without ambient light present was similar to the baseline scenario. However, the use of a blanket and the variation in shirt color led to a slightly larger mean RMSE and standard deviation of the of BRR value. This could be attributed to the increased noise introduced by the fabric folding during the respiratory motion. In this study, only a thin blanket was assessed, and a thicker blanket might further dampen the respiration movement, leading to worse results. Regarding shirt color, the higher mean and high standard deviation are noticeable. Green, gray and pink shirt colors were tested. Since the FPP technology relies on the reflection of the light on the subject’s surface, there may be a correlation between the darkness of the shirt color and its performance. Further investigations are needed to precisely characterize the impact, as this study only tested three different colors.

### 3.3. Research Findings

After accessing different scenarios, this research leads to the following findings:

- The baseline case demonstrated the successful extraction of a continuous respiration signal and the respiratory features RR and BRR with minor deviations from the reference. Varying the respiration modes did not significantly increase the deviations.
- Altering the subject’s position revealed high correlation for the prone and the lateral positions with more than 80% of all values within 1 bpm deviation of the reference both RR and BRR values.
- Turning off ambient light had no major impact on the results. Wearing a different shirt color or using a thin blanket still resulted in high correlation for the continuous respiration signal and a low RMSE for the extracted RR values. However, for the extracted BRR values, a higher standard deviation and mean of the RMSE were observed. Even in these situations, the BRR values remained within 1 bpm deviation from the reference in 90% of the cases.
- The system demonstrated robustness against slight body movements. For significant body movements, the body motions could be detected and marked but could not be reconstructed in all cases. Maintaining the RR and BRR values at a constant when the binary trust status was low resulted in errors during that period. The system reached its limit when the subject held its breath and moved simultaneously with a similar amplitude as respiratory movement.

## 4. Conclusions

In this paper, we developed, implemented and evaluated a respiration monitoring system based on FPP. The system comprised the 3D sensor prototype and the analysis algorithm to extract the respiratory parameters such as the respiration signal, RR, and BRR from the 3D images. An overall high mean correlation of 0.95 between the measured respiration signal and the reference signal was observed. The extracted RR values had a mean RMSE of 0.11 bpm to the reference values, while the BRR values showed a mean RMSE of 0.52 bpm to the reference. These values were obtained from various scenarios assessed to evaluate the system’s robustness. The proposed method proved to be robust toward slight body movements, but it was found to be less robust when significant body movements were present. Further research is required to ensure a reliable reconstruction of the respiratory signal in all cases. The high mean correlation of 0.95 to the reference indicates the clinical relevance of the proposed method.

## Figures and Tables

**Table 1:** Specifications of the projector used in our 3D sensor [37].

Attribute	Value
Model name	DLP Lightcrafter 4500
Vendor	Texas Instruments
Brightness	150 Lumens
F-number	2.1
Throw ratio	1.2
Aspect ratio	1.6
Resolution	912 × 1140
Mirror arrangement	Diagonal
Pixel size	7.6 μm
Maximum binary pattern rate	4225 Hz

**Table 2:** Specifications of the camera used in our 3D sensor [38].

Attribute	Value
Model name	GS3-U3-23S6C
Vendor	Point Grey Research (Now FLIR)
Sensor	Sony IMX174 (1/1.2${}^{\prime \prime }$ Color CMOS)
Maximum resolution	1920 × 1200
Corresponding frame rate	163 FPS
Lens mount	C-Mount
Pixel size	5.86 μm

**Table 3:** Geometrical values for the setup of the 3D sensor system.

Name	Variable	Value
Focal length	*f*	6 mm
Camera view short	$s_{c}$	433 mm
Camera view long	$l_{c}$	577.3 mm
Projector view short	$s_{p}$	484.2 mm
Projector view long	$l_{p}$	774.7 mm
Distance object plane	$d_{e}$	929.6 mm

**Table 4:** Specifications of the spirometer used as a reference system [47].

Attribute	Value
Model name	Go Direct Spirometer GDX-SPR
Vendor	Vernier Software & Technology
Resolution pressure sensor	0.02 Pa
Sampling rate	50 Hz

**Table 5:** Summary of evaluation scenarios.

Scenario	Description
Baseline	The subject was laying in supine position, ambient light was turned on and the subject was wearing a white shirt. The subject was asked to lay as still as possible and to breathe normally without conscious thought.
Fast breathing	The subject was asked to breathe as fast as possible. Not all of them were able to breathe fast for a whole minute; therefore, the dataset had to be shortened in some cases.
Hold breath	The subject was asked to hold their breath for a certain period within the 60 s measurement.
Deep chest breathing	The subject was asked to breathe in deeply and focus on thoracic breathing.
Deep abdominal breathing	The subject was asked to breathe in deeply and focus on diaphragm breathing.
Slight movement	The subject was asked to move around the upper body lightly. Actions were for, e.g., improving the position for comfort and moving the shoulders.
Significant movement	The subject was asked to move around more severely in all degrees of freedom.
Alternating shirt color	The subject was asked to wear a shirt of a different color than white.
Ambient light turned off	All ambient light sources in the room were turned off.
Lateral position	The subject was asked to lie on their side.
Prone position	The subject was asked to lie on their stomach.
Blanket usage	The subject was covered with a white blanket.

**Table 6:** Experimental results of the correlation coefficient (cc), the RR RMSE and the BRR RMSE (in bpm) for each scenario. The mean μ and the standard deviation σ are given across all participants of this study.

Scenario	$\mu_{\mathrm{cc}}$	$\sigma_{\mathrm{cc}}$	$\mu_{\mathrm{RR}}$	$\sigma_{\mathrm{RR}}$	$\mu_{\mathrm{BRR}}$	$\sigma_{\mathrm{BRR}}$
Baseline	0.974	0.016	0.048	0.027	0.236	0.136
Hold breath	0.965	0.022	0.033	0.015	0.437	0.177
Fast breathing	0.966	0.025	0.032	0.006	0.909	0.041
Deep abdomen	0.988	0.005	0.051	0.022	0.422	0.231
Deep chest	0.951	0.032	0.044	0.019	0.405	0.361
Prone position	0.876	0.167	0.017	0.008	0.489	0.438
Lateral position	0.941	0.034	0.517	0.336	0.875	0.137
Blanket used	0.916	0.069	0.078	0.054	0.446	0.149
No ambient light	0.95	0.062	0.074	0.062	0.198	0.032
Colored Tshirt	0.914	0.066	0.06	0.039	0.334	0.271
Slight movement	0.877	0.009	0.329	0.3	1.886	1.297
Significant movement	0.626	0.187	3.498	2.444	6.895	5.677

## Data Availability

The data presented in this study are available upon request from the corresponding author.

## Supplementary Materials

The following supporting information can be downloaded at: https://www.mdpi.com/article/10.3390/s23219000/s1.

## Abbreviations

The following abbreviations are used in this manuscript:

bpm	Breaths per minute	ICU	Intensive care unit
BRR	Breath-to-breath respiration rate	PSM	Phase-shifting method
FPP	Fringe projection profilometry	RMSE	Root-mean-square error
FPS	Frames per second	ROI	Region of interest
FOV	Field of view	RR	Respiration rate
MDPI	Multidisciplinary Digital Publishing Institute	SNR	Signal-to-noise ratio
NaN	Not a number	TKE	Taeger–Kaiser energy
NICU	Neonatal intensive care unit	ToF	Time-of-flight
